# A recyclable polyoxometalate-based supramolecular chemosensor for efficient detection of carbon dioxide†
†Electronic supplementary information (ESI) available. See DOI: 10.1039/c5sc02020d



**DOI:** 10.1039/c5sc02020d

**Published:** 2015-09-07

**Authors:** Haibing Wei, Jinlong Zhang, Nan Shi, Yang Liu, Ben Zhang, Jie Zhang, Xinhua Wan

**Affiliations:** a Beijing National Laboratory for Molecular Sciences , Key Laboratory of Polymer Chemistry and Physics of Ministry of Education , College of Chemistry and Molecular Engineering , Peking University , Beijing 100871 , China . Email: jz10@pku.edu.cn ; Email: xhwan@pku.edu.cn; b School of Chemistry and Chemical Engineering , Provincial Key Laboratory of Advanced Functional Materials and Devices , Hefei University of Technology , Hefei , Anhui 230009 , China

## Abstract

A new type of supramolecular chemosensor based on the polyoxometalate (POM) Na_9_DyW_10_O_36_ (DyW_10_) and the block copolymer poly(ethylene oxide-*b-N*,*N*-dimethylaminoethyl methacrylate) (PEO_114_-*b*-PDMAEMA_16_) is reported. By taking advantage of the CO_2_ sensitivity of PDMAEMA blocks to protonate the neutral tertiary amino groups, CO_2_ can induce the electrostatic coassembly of anionic DyW_10_ with protonated PDMAEMA blocks, and consequently trigger the luminescence chromism of DyW_10_ due to the change in the microenvironment of Dy^3+^. The hybrid complex in dilute aqueous solution is very sensitive to CO_2_ content and shows rapid responsiveness in luminescence. The luminescence intensity of the DyW_10_/PEO-*b*-PDMAEMA complex increases linearly with an increasing amount of dissolved CO_2_, which permits the qualitative and quantitative detection of CO_2_. The complex solution also shows good selectivity for CO_2_, with good interference tolerance of CO, N_2_, HCl, H_2_O and SO_2_. The supramolecular chemosensor can be recycled through disassembly of the hybrid complex by simply purging with inert gases to remove CO_2_.

## Abstract 2

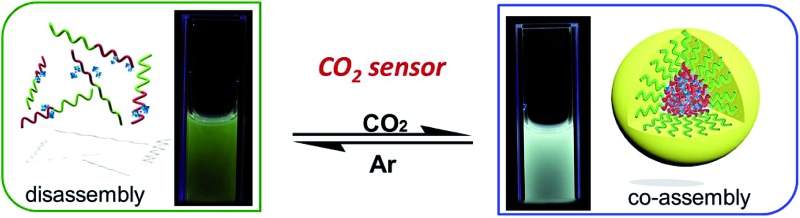
A new type of supramolecular chemosensor based on a polyoxometalate and a block copolymer was constructed for the qualitative and quantitative detection of CO_2_.

## Introduction

Carbon dioxide (CO_2_) is a known greenhouse gas which is responsible for global climate change and also related to many human diseases, such as hypercapnia, hypocapnia and metabolic disorders, as well as being important in coalmine safety and volcanic activity.^1^ CO_2_ sensing and detecting is of great significance. For example, monitoring of dissolved CO_2_ in arterial blood allows a timely clinical response in the case of patients with pneumonia or acute respiratory distress syndrome.^2^ Nowadays, CO_2_ detecting and sensing methods, including electrochemical systems,^3^ near-infrared spectroscopic techniques,^4^ gas chromatography^5^ and optical chemosensors^6^ are well-established. Among these, state-of-the-art Severinghaus-type electrochemical CO_2_ sensors are widely used in commercial clinical blood gas analyzers, but these probes still suffer from a long response time and are only capable of detecting a relatively high CO_2_ concentration, because their working performance relies on diffusion and the establishment of an equilibrium between the internal pH electrode and the sample.^7^ Alternatively, CO_2_ chemosensor systems based on colorimetric and fluorimetric analysis take advantage of the outcome of chromism visible to the naked eye, which can be helpful for rapid on-site monitoring.^8^ A few pH indicators or pH dependent fluorescent dyes have been used to produce such a type of optical sensor.^6a,8c–e^ However, pH-dependent organic chromatic molecules that can simultaneously meet the needs of appropiate p*K*_a_, specificity, photostability, and contrast are very limited.

Supramolecular chemosensors are promising candidates to overcome the limitations of conventional optical chemosensors. Specially designed supramolecular chemosensors are fabricated by noncovalent binding between the molecular recognizer and the molecular reporter, and the sensing works by a relay mechanism:^9^ the recognizer detects the analyte, then communicates with the reporter by physical or chemical means, and eventually the optical signals of the reporter are switched on. This strategy integrates the complementary functions of multiple components, and is advantageous for CO_2_ sensing in that it greatly expands the options of chromatic molecules and improves sensitivity and stability. In addition, by taking advantage of the dynamic nature of the noncovalent binding of supramolecular systems, device recyclability can be achieved, which is crucial for low cost and environmental concerns. Tang *et al.* ^8*b*^ developed a CO_2_ sensor based on a fluorogen with aggregation-induced emission in dipropylamine. Besides this, supramolecular CO_2_ sensors are still rare and it is a challenge to elaborately construct a supramolecular system for CO_2_ detection with the characteristics of high sensitivity, low detection limit, specificity, photostability, and recyclability.

**Scheme 1:**
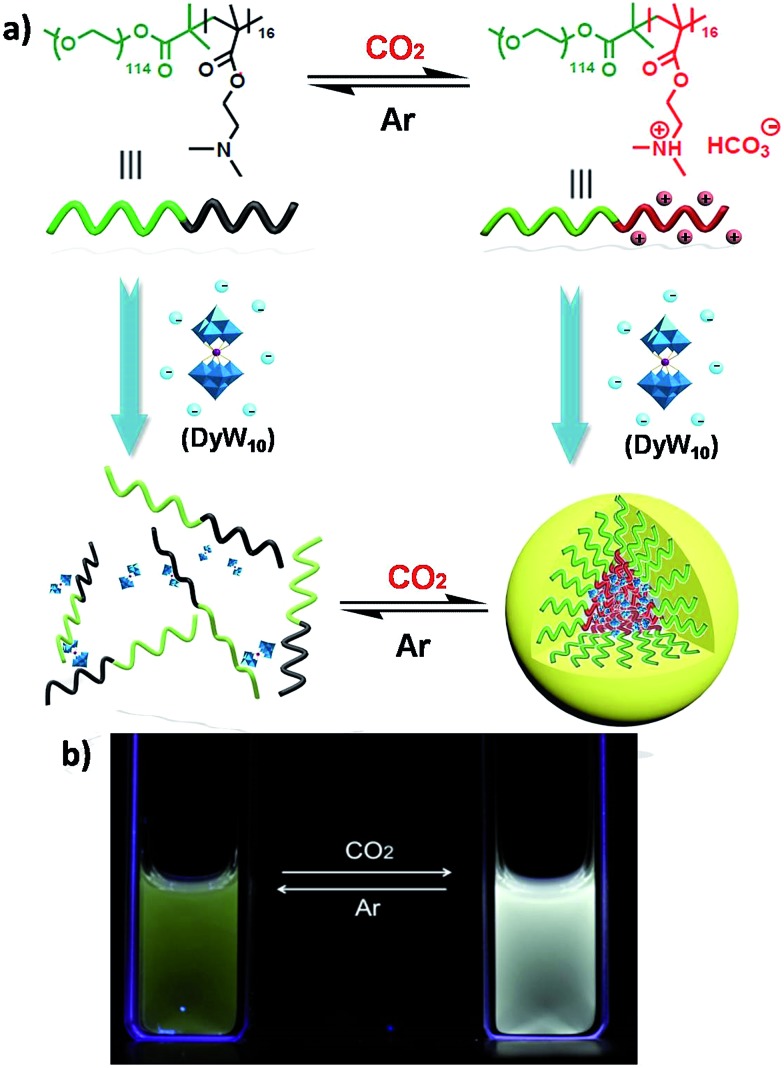
(a) Structural change of the PEO-*b*-PDMAEMA block copolymer and schematic representation of the reversible formation of a hybrid micelle after the reaction with CO_2_ in aqueous medium. (b) Photos taken under illumination with 254 nm UV light, representing the CO_2_-responsive luminescence chromism of the DyW_10_/PEO-*b*-PDMAEMA complex in aqueous solution before/after CO_2_ sensing.

Recently we have discovered hybrid supramolecular systems with the variable luminescence properties of lanthanide-containing polyoxometalates (POMs) by coacervate complexation with block polyelectrolytes.^10^ The lanthanide-containing polyoxometalates possess excellent photoluminescent properties, *i.e.* narrow emission bands, large Stokes shifts, long lifetimes and photostability, and are sensitive to ambient chemical environments.^11^ The dysprosium-containing POM Na_9_DyW_10_O_36_ (DyW_10_) has two characteristic emission bands, *viz.*, ^4^F_9/2_ → ^6^H_15/2_ (blue emission, *λ*maxem = 476 nm) and ^4^F_9/2_ → ^6^H_13/2_ (yellow emission, *λ*maxem = 574 nm) transitions, and their relative intensity ratio 
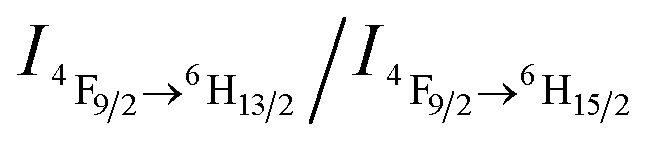
 varies according to the surrounding microenvironment, which results in luminescence chromism.^11a,12^ Although the luminescence of lanthanide-containing POMs is not sensitive to CO_2_, assembly approaches can effectively tune their emission colors and intensities, and therefore their characteristics are promising when employed as supramolecular sensors.^13^ In the present work, we constructed a DyW_10_-based supramolecular chemosensor for CO_2_ detection and quantitation in aqueous systems with high sensitivity and specificity, rapid response and recyclability. Unlike previous CO_2_ chemosensors, which depend primarily on chromism of organic fluorogens at the molecular level, our supramolecular CO_2_ sensor is based on hybrid core–shell assemblies composed of the block copolymer poly(ethylene oxide-*b-N*,*N*-dimethylaminoethyl methacrylate) (PEO_114_-*b*-PDMAEMA_16_) and DyW_10_ in aqueous solution. By taking advantage of the CO_2_ sensitivity of PDMAEMA blocks to protonate the neutral tertiary amino groups,^14^ CO_2_ can induce the electrostatic coassembly of anionic DyW_10_ with protonated PDMAEMA blocks, and consequently trigger the luminescence chromism of DyW_10_ due to the change in the microenvironment of Dy^3+^ (Scheme 1a). The luminescence variation is closely related to the CO_2_ content in solution, which can be used to quantitate dissolved CO_2_.

## Results and discussion

**Fig. 1:**
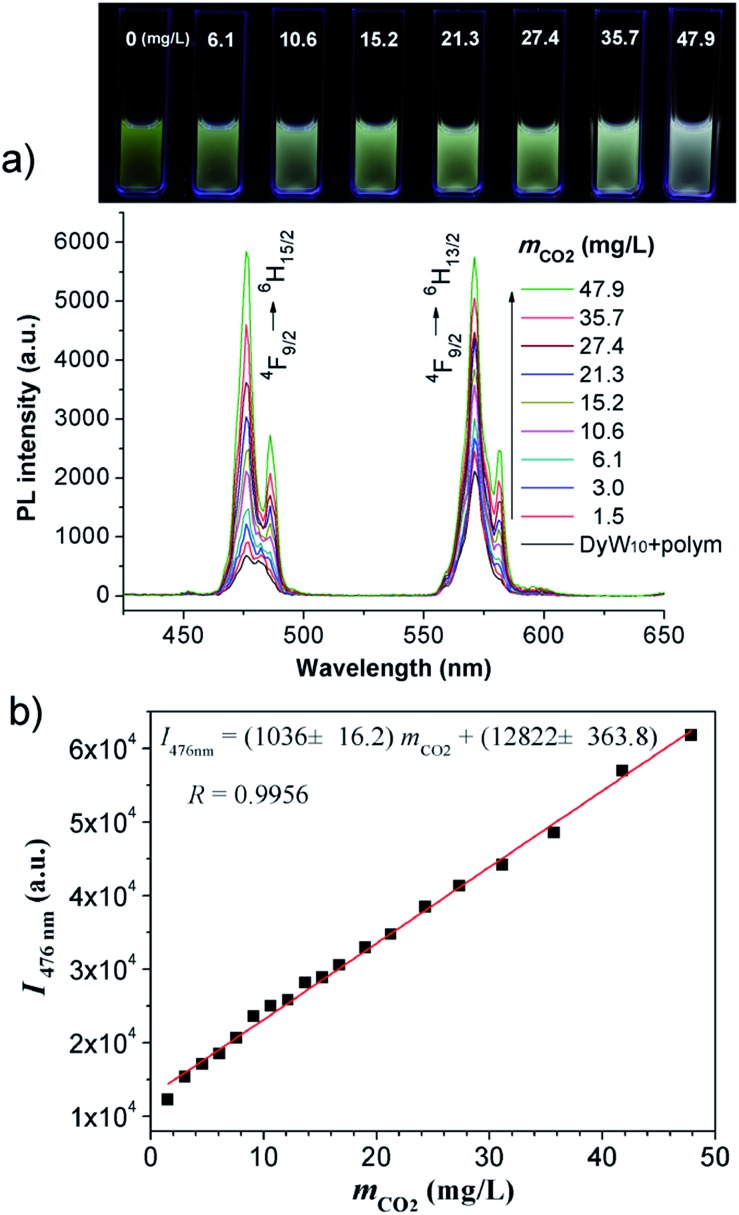
(a) Variation in the PL spectra of DyW_10_/PEO-*b*-PDMAEMA hybrid complex (DyW_10_: 0.2 mg mL^–1^, 9.8 mL) in the presence of various concentrations of dissolved CO_2_ at 25 °C (*λ*_ex_ = 280 nm). Insert: photograph of DyW_10_/PEO-*b*-PDMAEMA hybrid complex in aqueous solution with different concentrations of dissolved CO_2_ under UV illumination. (b) Plot of PL integrated intensities (blue emission, 
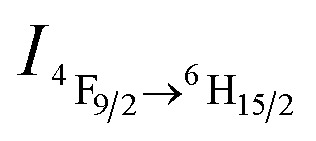
) of the DyW_10_/PEO-*b*-PDMAEMA hybrid complex in aqueous solution as a function of dissolved CO_2_ concentration at 25 °C (*λ*_ex_ = 280 nm).

The DyW_10_/PEO-*b*-PDMAEMA complex was prepared by the addition of PEO-*b*-PDMAEMA to a dilute DyW_10_ solution (0.2 mg mL^–1^, 9.8 mL), where the molar ratio of tertiary amino groups in PDMAEMA to DyW_10_ was set as 13.5 (the charge ratio of DyW_10_/PDMAEMA ∼1.0). Bubbling a small volume of CO_2_ gas through the solution in as short a time as <1 minute caused a striking change in the emission of DyW_10_ from weak green light to intense white light (Scheme 1b), while bubbling CO_2_ into dilute DyW_10_ solution for a long time did not cause a color change. The quantum yield of the DyW_10_/PEO-*b*-PDMAEMA complex increased from 0.78% to 2.10% after treatment with CO_2_, while the molar absorptivity was almost unchanged (∼7.0 × 10^3^ L mol^–1^ cm^–1^ on the basis of Na_9_DyW_10_O_36_). As a potential CO_2_ sensor, the complex solution is very sensitive to dissolved CO_2_ content and shows rapid responsiveness. To track the luminescence variation at low contents of dissolved CO_2_, a certain amount of saturated CO_2_ aqueous solution (1.45 g L^–1^ at 100 kPa and 25 °C) was directly added to the hybrid complex solution. *In situ* PL monitoring was conducted to investigate the sensing time of the CO_2_, and a substantial increase in the emission intensity was observed after only 30 s. As can be seen in Fig. 1a, the luminescence intensity of the DyW_10_/PEO-*b*-PDMAEMA complex increased linearly with increasing content of dissolved CO_2_ with a correlation coefficient of 0.9956 (Fig. 1b). As the value of 
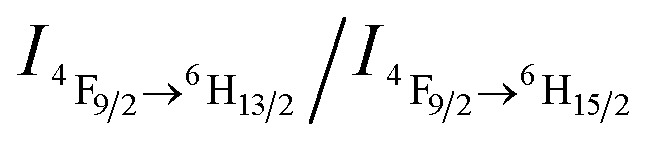
 decreased with increasing CO_2_ concentration (Fig. S1†), the emission color gradually evolved from green to white, as could be seen with the naked eye. The detection limit of dissolved CO_2_ was around 1.5 mg L^–1^. The DyW_10_/PEO-*b*-PDMAEMA sensor responds to dissolved CO_2_ in the range 0–47.9 mg L^–1^, and the linear range can be extended by changing the initial concentration of the DyW_10_/PEO-*b*-PDMAEMA complex (Fig. S2†). Therefore, this photophysical characteristic of DyW_10_/PEO-*b*-PDMAEMA solution allows for the qualitative and quantitative detection of CO_2_. Moreover, upon exposure to air, atmospheric CO_2_ (∼300 ppm) can stimulate the luminescence variation of the DyW_10_/PEO-*b*-PDMAEMA complex system (Fig. S3†), further demonstrating its high sensitivity.

The complex solution shows good selectivity for CO_2_. CO, HCl, and SO_2_ gases were purged into the solution. CO did not change the luminescence at all (Fig. S4†), while SO_2_ quenched the luminescence because of the reduction of DyW_10_ (Fig. S5†) and HCl gas also quenched the luminescence at low pH values (Fig. S6†) owing to the decomposition of DyW_10_. However, after purging with a mixed gas containing 20% CO_2_, 70% N_2_, 10% O_2_ and 0.1% SO_2_, the complex solution still showed good detection of CO_2_ (Fig. S7†), indicating that in practical applications CO, SO_2_ and O_2_ would not affect the CO_2_ detection.

**Fig. 2:**
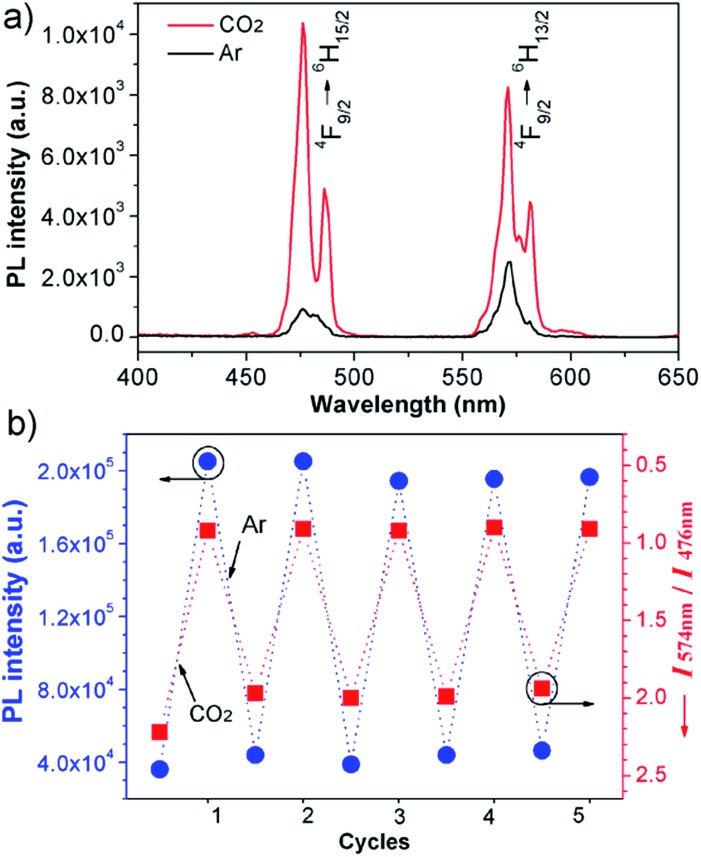
(a) Emission spectra (*λ*_ex_ = 280 nm) of DyW_10_/PEO-*b*-PDMAEMA coassembly in water before and after CO_2_ treatment. (b) Reversible switching of the luminescence intensity and chromism of the DyW_10_/PEO-*b*-PDMAEMA solution by alternating CO_2_/Ar treatment.

Furthermore, the sensor can be recycled by purging with inert gas to degas CO_2_. As shown in Fig. 2a, the emission spectrum of the complex solution after CO_2_ treatment displays two intense blue and yellow bands, with an 
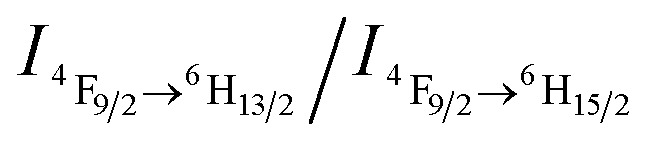
 value of ∼0.92. After purging with Ar to degas CO_2_, the intensity strikingly decreased to the initial value before CO_2_ treatment. 
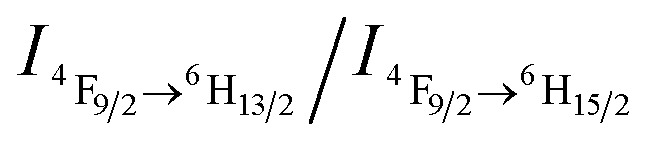
 also increased to ∼2.22, and correspondingly the emissive color recovered from strong white to the original weak green. Aside from Ar, the luminescence can be restored by purging with N_2_ or simply heating, although the heating treatment takes longer (Fig. S8 and S9†). Unlike Ar and N_2_, compressed air is inefficient (Fig. S10†), which may be attributed to the fact that the air contains a small amount of CO_2_ (∼300 ppm). On the other hand, the complex solution in both states is very stable in an airtight environment, with no distinct changes after standing at room temperature for at least 30 h (Fig. S11†). Both the luminescence intensity and emission color of the DyW_10_/PEO-*b*-PDMAEMA complex can be reversibly switched by alternating CO_2_/Ar treatment for at least five cycles (Fig. 2b), which endows this complex system with the merit of recyclability, making it a more affordable CO_2_ detector.

**Fig. 3:**
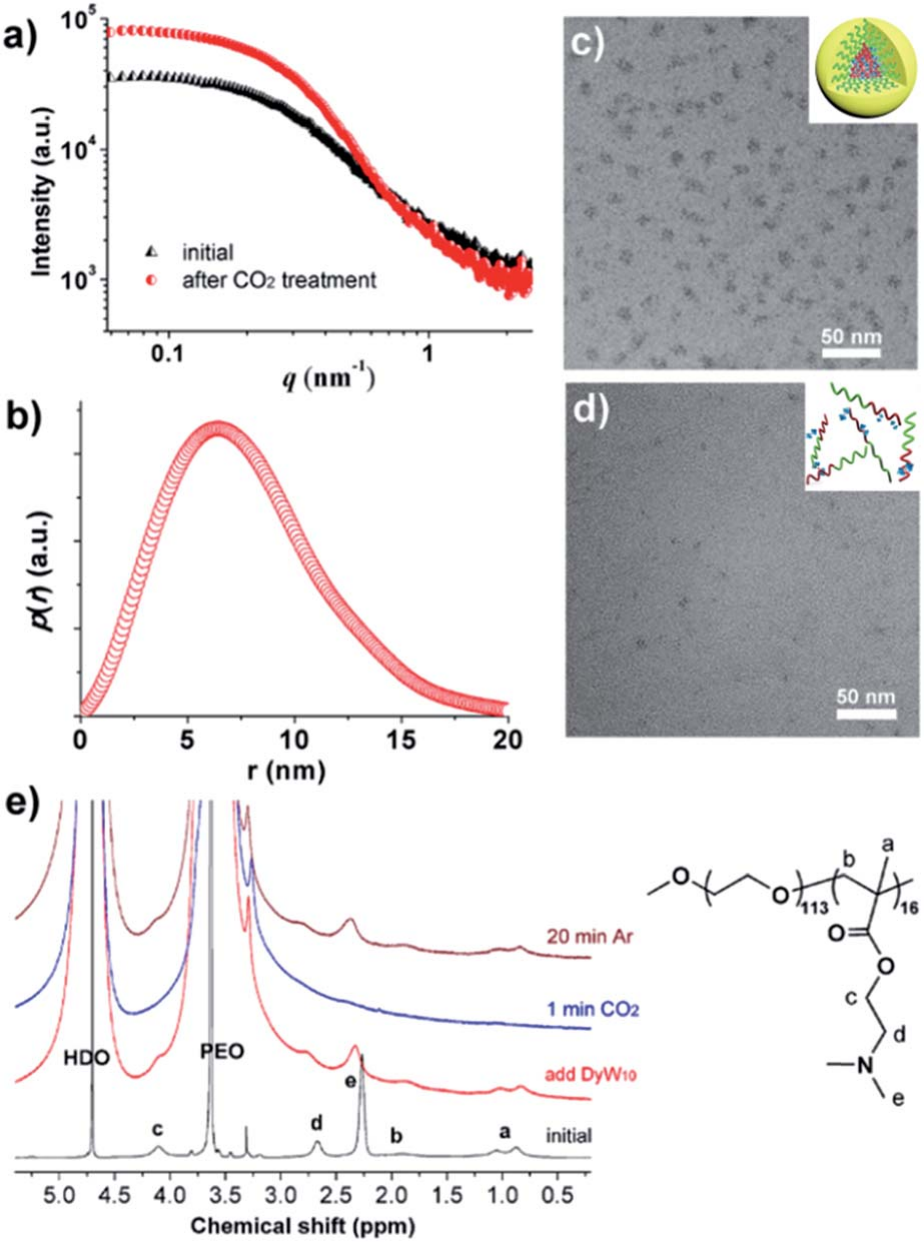
Characterization of the morphology of the DyW_10_/PEO-*b*-PDMAEMA coassemblies before and after CO_2_ treatment. (a) The SAXS pattern obtained for DyW_10_/PEO-*b*-PDMAEMA and (b) the corresponding distance distribution, *p*(*r*), after treatment with CO_2_; TEM images of DyW_10_/PEO-*b*-PDMAEMA coassemblies after CO_2_ (c) and Ar (d) treatment; (e) partial ^1^H NMR spectra in D_2_O recorded for the DyW_10_/PEO-*b*-PDMAEMA complex, followed by purging with CO_2_ gas, and then degassing CO_2_ with Ar.

To verify the CO_2_-responsive assembly behavior of DyW_10_/PEO-*b*-PDMAEMA, we used *in situ* small angle X-ray scattering (SAXS), transmission electron microscopy (TEM), and ^1^H NMR spectroscopy. The scattering intensity *I*(*q*) of the DyW_10_/copolymer complex at low *q* values became stronger after purging CO_2_ into the complex solution (Fig. 3a), supporting the formation of large assembled aggregates. The corresponding pair-distance distribution function *p*(*r*) (Fig. 3b) was deduced by using Generalized Indirect Fourier Transform (GIFT) analysis,^15^ and the result shows that the scattering objects have a globular, almost spherical shape with an average diameter of ∼12 nm. The TEM image of DyW_10_/PEO-*b*-PDMAEMA assemblies after CO_2_ treatment displays spherical micelles with an average diameter of 10 nm with a narrow distribution (Fig. 3c), in good agreement with the SAXS results, while before CO_2_ treatment the TEM image displays a lesser amount of small irregular aggregates (Fig. 3d). To probe the PDMAEMA segments participating in the formation of a micellar core with the macroanionic DyW_10_, ^1^H NMR spectroscopy was utilized to characterize the signal changes of the PDMAEMA segments in D_2_O solution (Fig. 3e). As expected, compared to the characteristic signals of PDMAEMA at chemical shifts ∼2.38, 2.81 and 4.15 ppm recorded for the initial solution, the PDMAEMA signals disappeared completely after CO_2_ treatment, indicating that almost all PDMAEMA segments participate in the formation of the coacervate core. Furthermore, the ^1^H NMR signals of the PDMAEMA blocks can be restored upon treatment with Ar. All the above results support the co-assembly of DyW_10_ and PEO-*b*-PDMAEMA after CO_2_ treatment into dense spherical micelles, and the fact that the assembly/disassembly can be reversibly switched by alternating CO_2_/Ar treatment.

To explore the microenvironment variations of the luminophore DyW_10_ before and after purging with CO_2_, further examination of the decay lifetimes of DyW_10_/PEO-*b*-PDMAEMA coassemblies was carried out (Fig. S12†). It is known that the emission of Dy^3+^ is highly dependent on coordinated water, due to radiationless deactivation of the ^4^F_9/2_ excited state through weak coupling with the vibrational states of the high-frequency OH oscillators in the water ligands.^16^ In the initial state without CO_2_, three lifetimes could be identified: *τ*_1_ ≈ 4.0 μs (*f*_1_ = 0.366), *τ*_2_ ≈ 17.3 μs (*f*_2_ = 0.201), and *τ*_3_ ≈ 59.9 μs (*f*_3_ = 0.433) (*f*_*i*_ denotes the fractional contribution to the total fluorescence decay; detailed fitting methods and results are available in Table S1†).^17^ As the lifetime is correlated to the water molecules coordinated to the Dy^3+^ ion, the number of water ligands *q*_H_2_O_ can be estimated to be about 6.2, 1.2, and 0.16, respectively,^18^ demonstrating that there are a considerable number of water molecules coordinated to Dy^3+^ in the initial state (detailed calculation of *q*_H_2_O_ is given in the ESI†). In comparison, after addition of CO_2_, there is only a single long decay lifetime of ∼57.5 μs with a *q*_H_2_O_ value of 0.18, which is comparable to that of DyW_10_ crystals (*τ* ≈ 58.2 μs, *q*_H_2_O_ ∼0.17), indicating there are almost no water molecules coordinated to the Dy^3+^ ion. This result further suggests that after CO_2_ treatment DyW_10_ is located in a relatively hydrophobic environment in the complex core of spherical micelles, where the cationic PDMAEMA segments have strong enough electrostatic affinity to the anionic DyW_10_ to replace the water ligands.

What follows is a proposed mechanism of how CO_2_ triggers the luminescence chromism. In the initial DyW_10_/PEO-*b*-PDMAEMA dilute solution, the degree of protonation of the PDMAEMA blocks is estimated to be ∼61% based on the initial pH value ∼7.20 of the DyW_10_/PEO-*b*-PDMAEMA solution (see ESI†). The water molecules coordinated to Dy^3+^ are only partially replaced by PDMAEMA segments through electrostatic interactions, which in fact lowers the *D*_4d_ symmetry of DyW_10_ in the solid to *C*_4v_, because the water molecules cannot lie exactly in the reflection plane of the alternating *S*_8_ axis. The 
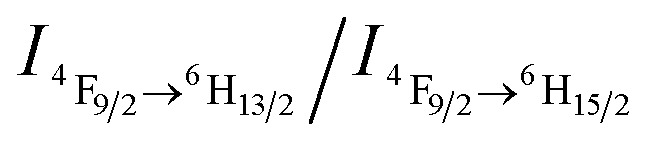
 value ∼2.22 as a probe of Dy^3+^ symmetry in ambient microenvironments also demonstrates that DyW_10_ is located in a relatively asymmetrical microenvironment. In the presence of CO_2_, the *N*,*N*-dimethylaminoethyl tertiary amino groups of PEO-*b*-PDMAEMA are almost completely converted to positively-charged ammonium bicarbonates (Scheme 1), and the PDMAEMA block is almost fully positively charged with *δ* ∼ 99.8% as estimated from the pH value ∼4.80. Consequently, the electrostatic interactions between the cationic copolymer and macroanionic DyW_10_ are greatly enhanced and drive their co-assembly into dense spherical micelles consisting of a hydrophobic PDMAEMA/DyW_10_ complex core stabilized by a corona of neutral hydrophilic PEO blocks. As DyW_10_ is located in the dense core of the micelles, its bound water molecules are almost completely replaced by the protonated PDMAEMA segments, and thus the symmetric microenvironment of DyW_10_ is improved as evidenced by a decrease in the 
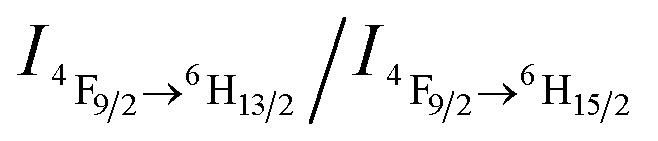
 value to 0.92, which is comparable to that of DyW_10_ crystals. As a result, the luminescence intensity is greatly enhanced and the chromism from green to white occurs. On purging with Ar to remove CO_2_, the spherical micelles can disassemble because of the partial deprotonation of PDMAEMA, and consequently the complex solution can be used for recyclable CO_2_ sensing. Its performance does not decline with the number of cycles, because the CO_2_/Ar switching does not cause any salt accumulation or contamination which would destroy the electrostatic assemblies.

## Conclusions

In summary, we have demonstrated a novel supramolecular assay for fluorimetric sensing of carbon dioxide based on a POM/copolymer hybrid complex. PDMAEMA blocks could be protonated by CO_2_ leading to electrostatic co-assembly with DyW_10_, and consequently the white emission of DyW_10_ is switched on. The fluorimetric characteristics of DyW_10_/PEO-*b*-PDMAEMA coassemblies permit the detection of CO_2_ with the merits of simplicity, sensitivity, specificity, interference tolerance, and recyclability. Furthermore, our findings may pave the way to the elaborate design of smart supramolecular materials with complementary functional components.
