# Supplementary material for: A recyclable polyoxometalate-based supramolecular chemosensor for efficient detection of carbon dioxide
Source: Chem Sci. 2015 Sep 7;6(12):7201–5. doi: 10.1039/c5sc02020d (PMC5512013; doi:10.1039/c5sc02020d)
Supplement: Supplementary file 1 [file SC-006-C5SC02020D-s001.pdf]

Electronic Supplementary Material (ESI) for Chemical Science.
This journal is © The Royal Society of Chemistry 2015

Electronic Supplementary Information

for

A Recyclable Polyoxometalate-based Supramolecular Chemosensor for Efficient Detection of Carbon Dioxide

Haibing Wei,[ab] Jinlong Zhang,[a] Nan Shi,[a] Yang Liu,[a] Ben Zhang,[a] Jie Zhang,*[a] and Xinhua Wan*[a]

[a] Beijing National Laboratory for Molecular Sciences, Key Laboratoryof Polymer Chemistry and Physics of Ministry of Education, Collegeof Chemistry and Molecular Engineering, Peking University, Beijing 100871, China
[b] School of Chemistry and Chemical Engineering, Provincial Key Laboratory of Advanced Functional Materials and Devices, Hefei University of Technology, Hefei, Anhui 230009, China.

*To whom correspondence should be addressed.
E-mail: jz10@pku.edu.cn (J. Z.) and xhwan@pku.edu.cn (X.H. W.)

Experimental Section

Materials and Instruments. $Na_9DyW_{10}O_{36}$ was prepared as described by Caronado.[S1] Block copolymer PEO_{114}-*b*-$PDMAEMA_{16}$ (PDI=1.2) was purchased from Polymer Source, Canada. The deprotonation of PEO_{114}-*b*-$PDMAEMA_{16}$ was carried out by adjusting its aqueous solution with NaOH to pH = 12, and then extracting with CH_2Cl_2, finally precipitated in *n*-hexane/ethyl ether (v/v, 7/3). All aqueous solutions of polyoxometalate and polymers were prepared using ultrapure water from a Millipore Milli-Q system. CO_2 (99.99%) and argon (99.999%) were purchased from Beijing Haike Yuanchang general aeriform Co. Ltd and used as received.

^1H NMR spectra were recorded on a Bruker 400 AVANCE III spectrometer operating at 400.23 MHz, using D_2O as solvent. TEM images were obtained on a JEM 2100 instrument operating at an acceleration voltage of 200 kV. The specimen was prepared by drop-casting the sample solution onto a carbon-coated copper grid for a few minutes. Excess solution was blotted away with a strip of filter paper, and the sample grid was dried in air. Photoluminescence measurements were performed on an FLS 920 Steady State & Time-resolved Fluorescence Spectrometer (Edinburgh Instruments Ltd.). Small-angle X-ray scattering (SAXS) measurements were performed using a SAXSess (Anton Paar) equipped with Kratky block-collimation system. The scattering pattern was recorded on an imaging plate (IP) with a pixel size of 42.3×42.3 μm^2 which extended to the high-angle range (the q range covered by the IP was from 0.06 to 29 nm^{-1}). We carefully loaded the DyW_{10}/PEO-*b*-PDMAEMA solution samples before and after exposure to CO_2 and upon Ar bubbling that removes CO_2 for SAXS measurement into a quartz capillary with a diameter of 1 mm at ambient temperature. The scattering curve of pure water filled in the same capillary was measured as the background. Background subtraction and desmearing were conducted by using SAXSquant 3.6 software. The pairdistance distribution function (PDDF) of scattering curves was calculated using the generalized indirect Fourier transform (GIFT)[S2] program included in the SAXSess software package. The

quantum yields of the complex solution were determined by the relative method with tryptophan as standard, excited at 280 nm.

Calculation of protonation degree of DMAEMA in the copolymer PEO_{114}-*b*-$PDMAEMA_{16}$

The pH change was measured using a pH-meter (Mettler). The protonation equation of DMAEMA can be expressed as follows:

$$\text{PDMAEMA} + [\text{H}^+] \rightleftharpoons \text{PDMAEMAH}^+$$

The degree of protonation (δ) of DMAEMA in diblock copolymer PEO-*b*-PDMAEMA mainly depends on its pK_a. As many reports mentioned, the pK_a of PDMAEMA was approximately 7.4.[S3] Therefore, the quantity of the protonated DMAEMA in every PEO-*b*-PDMAEMA chain at different pHs can be calculated according to eqs. (1), (2), and (3):

$$K_a = \frac{[\text{DMAEMA}] \bullet [\text{H}^+]}{[\text{DMAEMAH}^+]} \quad (1)$$

$$\text{p}K_a = \text{pH} + \log_{10} \frac{[\text{DMAEMAH}^+]}{[\text{DMAEMA}]} \quad (2)$$

$$\delta = \frac{[\text{DMAEMAH}^+]}{[\text{DMAEMA}] + [\text{DMAEMAH}^+]} \quad (3)$$

where [DMAEMA] and [DMAEMAH$^+$] are the concentration of non-protonated DMAEMA molecules and protonated DMAEMA molecules in copolymer PEO-*b*-PDMAEMA, respectively. The pH value of initial DyW_{10}/PEO-*b*-PDMAEMA complex solution was about 7.2, and the δ can be calculated to be 61%. Upon alternative treatment with CO_2 and Ar, the pH of DyW_{10}/PEO-*b*-PDMAEMA complex solution can be switched between 4.8 and 6.9. Thus, δ can be calculated to be 99.8% at pH 4.80 and 75.1% at pH 6.92.

Fluorescence lifetime analysis

The fluorescence decay measurements were performed using a time-correlated single-

photon counting technique, in which the fluorescence decay was monitored at 476 nm. The fluorescence decays were fitted by the following equation:

$$I(t) = \sum f_i \exp(-t/\tau_i)$$

where f_i represents fractional contribution to the total fluorescence decay.[S4] In DyW_{10} aqueous solution (0.2 mg mL^{-1}), the DyW_{10} luminescence showed first-order decay with a lifetime 5.2 μs. With the addition of PEO-*b*-PDMAEMA, the decay curves cannot be fitted by a single exponential but at least three lifetimes, where the lifetime was much longer. In addition, excellent first-order decay was turned up as CO_2 gas bubbled into the DyW_{10} aqueous solution in the presence of PEO-*b*-PDMAEMA, where the lifetime is much longer, as to 57.5 μs. Interestingly, after degassing CO_2 dissolved in the solution by purging Ar, the decay lifetimes was restored to three lifetimes, that is, $\tau_1 \approx 4.5$ μs, $\tau_2 \approx 20$ μs, and $\tau_3 \approx 60$ μs, which is similar to the initial state of DyW_{10}/PEO-*b*-PDMAEMA complex. The fitting parameters of each state are summarized in Table S1.

Calculation the number of water molecules (*q*) coordinated to Dy^{3+} ion

The values of decay lifetime of DyW_{10}/PEO-*b*-PDMAEMA complex in H_2O solution and its corresponding D_2O solution were recorded. According to the literature,[S5] the number of water molecules *q* coordinated at the dysprosium center can be calculated by using the following equation:

$$q = 2.61 \times 10^{-2} \left(\tau_{\mathrm{H_2O}}^{-1} - \tau_{\mathrm{D_2O}}^{-1}\right)$$

where $\tau_{H_2O}^{-1}$ and $\tau_{D_2O}^{-1}$ are the reciprocal experimental lifetimes in H_2O and D_2O solutions in ms^{-1}, respectively. For example, the experimental lifetime of DyW_{10} in H_2O and D_2O solutions (0.2 mg mL^{-1}) is 0.0053 and 0.0954 ms, respectively. Therefore, the *q* value was calculated to be 4.6, indicating that there are probably four water molecules coordinated to Dy^{3+} ion. Likewise, the experimental lifetimes of DyW_{10}/PEO-*b*-PDMAEMA complex in H_2O and D_2O solutions upon with CO_2 treatment is 0.06 and 0.1 ms, respectively, therefore, the *q* value was calculated to be

0.18, indicating that probably no water molecules was coordinated to Dy^{3+} ion.

References:

S1. AlDamen, M. A.; Cardona-Serra, S.; Clemente-Juan, J. M.; Coronado, E.; Gaita-Arino, A.; Marti-Gastaldo, C.; Luis, F.; Montero, O. *Inorg. Chem.* **2009,** *48*, 3467.

S2. (a) Lof, D.; Tomsic, M.; Glatter, O.; Fritz-Popovski, G.; Schillen, K. *J. Phy. Chem. B* **2009**, *113*, 5478. (b) Glatter, O.; Fritz, G.; Lindner, H.; Brunner-Popela, J.; Mittelbach, R.; Strey, R.; Egelhaaf, S. U. *Langmuir* **2000**, *16*, 8692. (c) Weyerich, B.; Brunner-Popela, J.; Glatter, O. *J. Appl. Crystallogr.* **1999**, *32*, 197.

S3. (a) Bütün, V.; Armes, S. P.; Billingham, N. C. *Polymer* **2001,** *42*, 5993. (b) Fournier, D.; Hoogenboom, R.; Thijs, H. M. L.; Paulus, R. M.; Schubert, U. S. *Macromolecules* **2007,** *40*, 915.

S4. Lakowicz, J. R., *Principles of Fluorescence Spectroscopy, 3rd. ed.; Springer: Berlin,* **2006**, 101-103.

S5. Hall, C. D.; Sharpe, N. W. *J. Photochem. Photobio. A: Chem.* **1990**, *52*, 363.

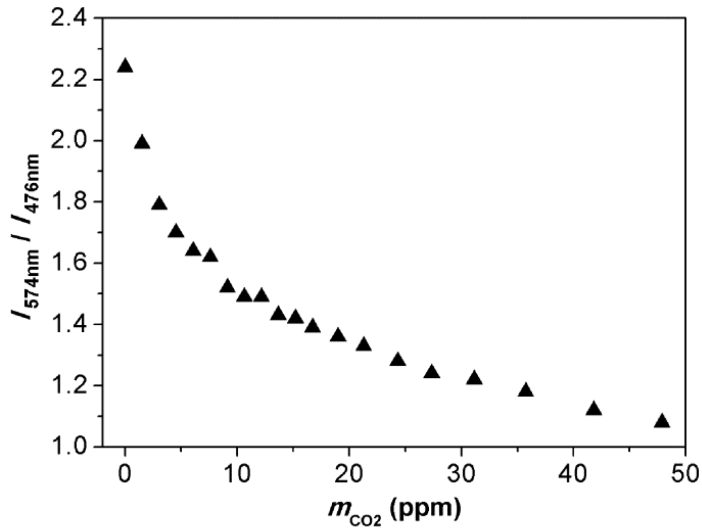

Figure S1. Variation of $I_{4F9/2\rightarrow6H13/2}/I_{4F9/2\rightarrow6H15/2}$ values in the PL spectra of DyW_{10}/PEO-*b*-PDMAEMA hybrid complex in aqueous solution as a function of the concentration of CO_2 at 25 °C (λ_{ex} = 280 nm).

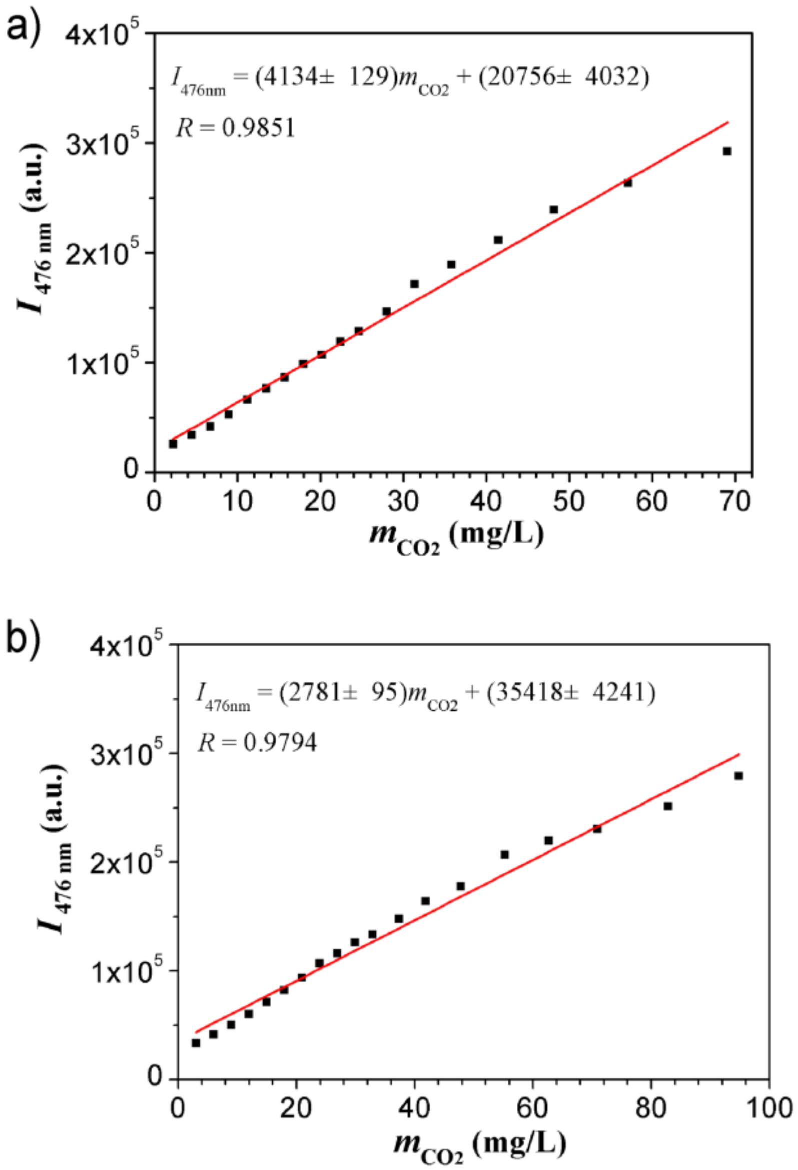

Figure S2. Plots of PL intensities (blue emission, $I_{4F9/2 \rightarrow 6H15/2}$) of DyW_{10}/PEO-*b*-PDMAEMA complex with DyW_{10} concentration of **(a)** 0.3 mg/mL and **(b)** 0.4 mg/mL in aqueous solutions a function of dissolved CO_2 concentrations at 25 °C (λ_{ex} = 280 nm).

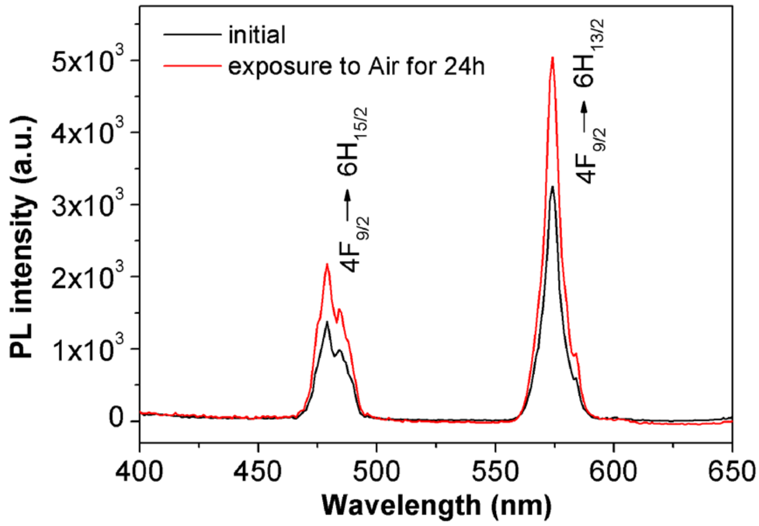

Figure S3. The emission spectra of DyW_{10}/PEO-*b*-PDMAEMA hybrid complex solution before and after exposure to air for 24 h at room temperature (λ_{ex} = 280 nm)

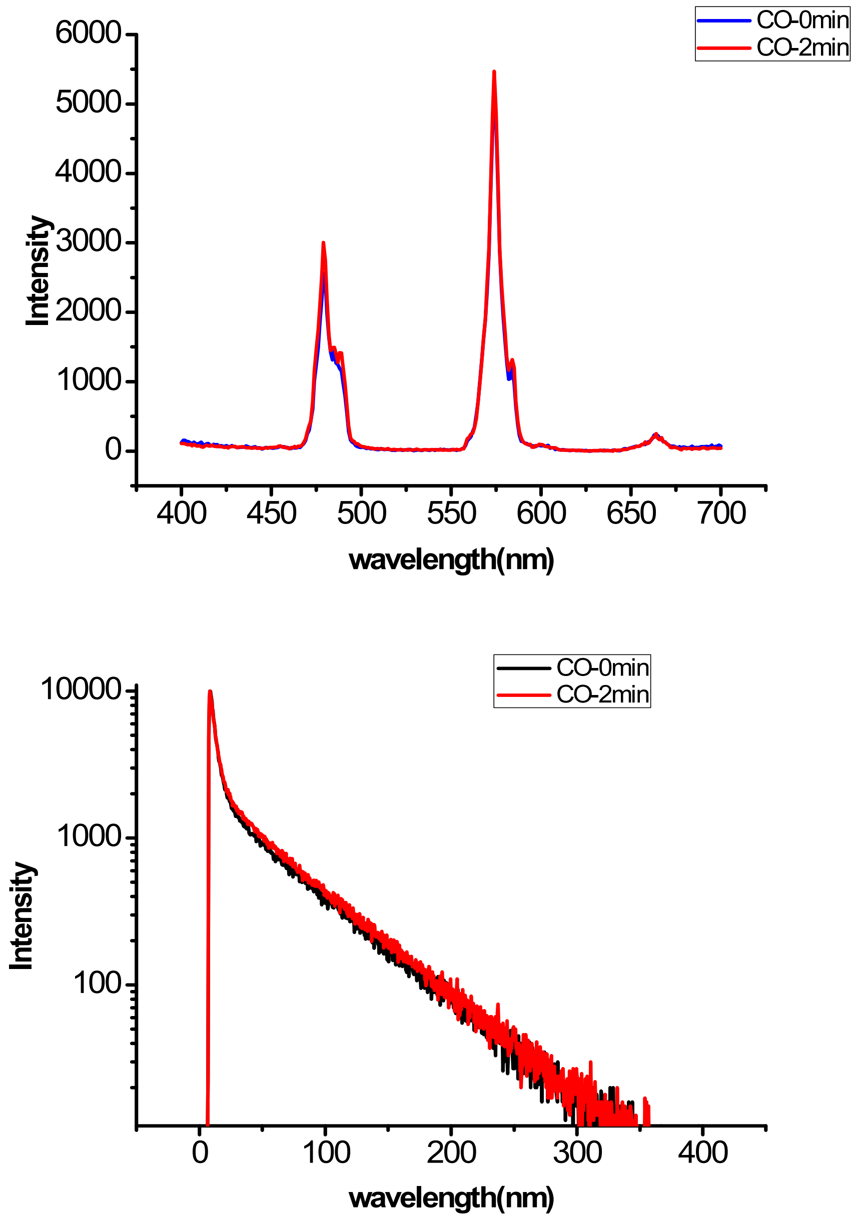

Figure S4. The emission spectra (up) and decay curves (down) of DyW_{10}/ PEO_{113}-*b*-$PDMAEMA_{16}$ complex solution before and after purging CO for a couple of minutes at 25 °C (λ_{ex} = 280 nm)

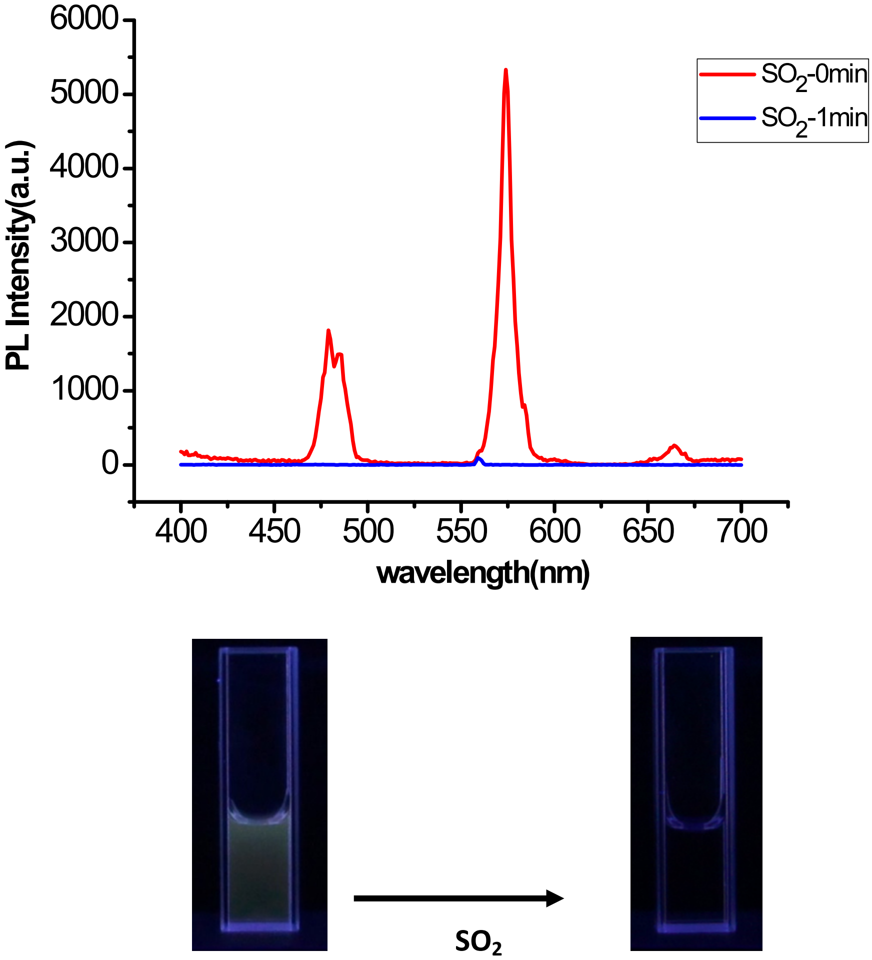

Figure S5. The emission spectra (up) and the corresponding photograph (down) of DyW_{10}/ PEO_{113}-*b*-$PDMAEMA_{16}$ hybrid complex solution before and after purging SO_2 for a couple of minutes at 25 °C (λ_{ex} = 280 nm)

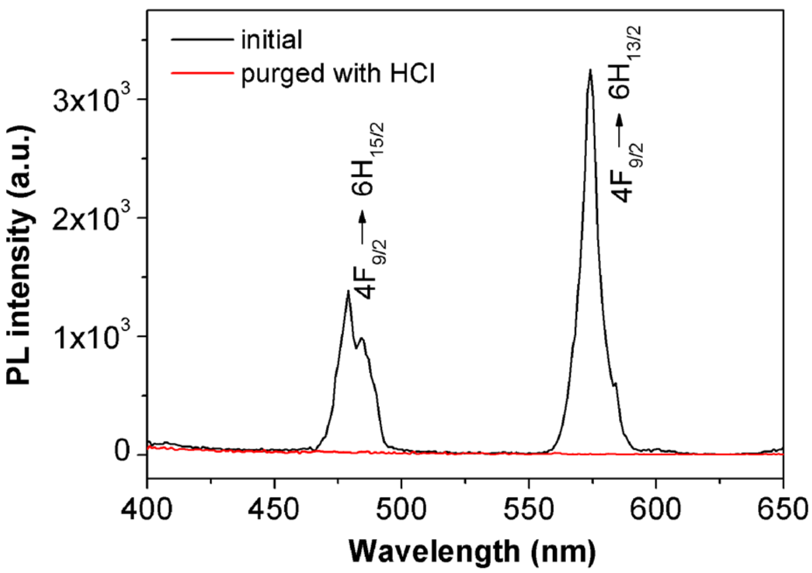

Figure S6. The emission spectra of DyW_{10}/ PEO-*b*-PDMAEMA hybrid complex solution before and after purging with the HCl gas for a couple of minutes at 25 °C (λ_{ex} = 280 nm)

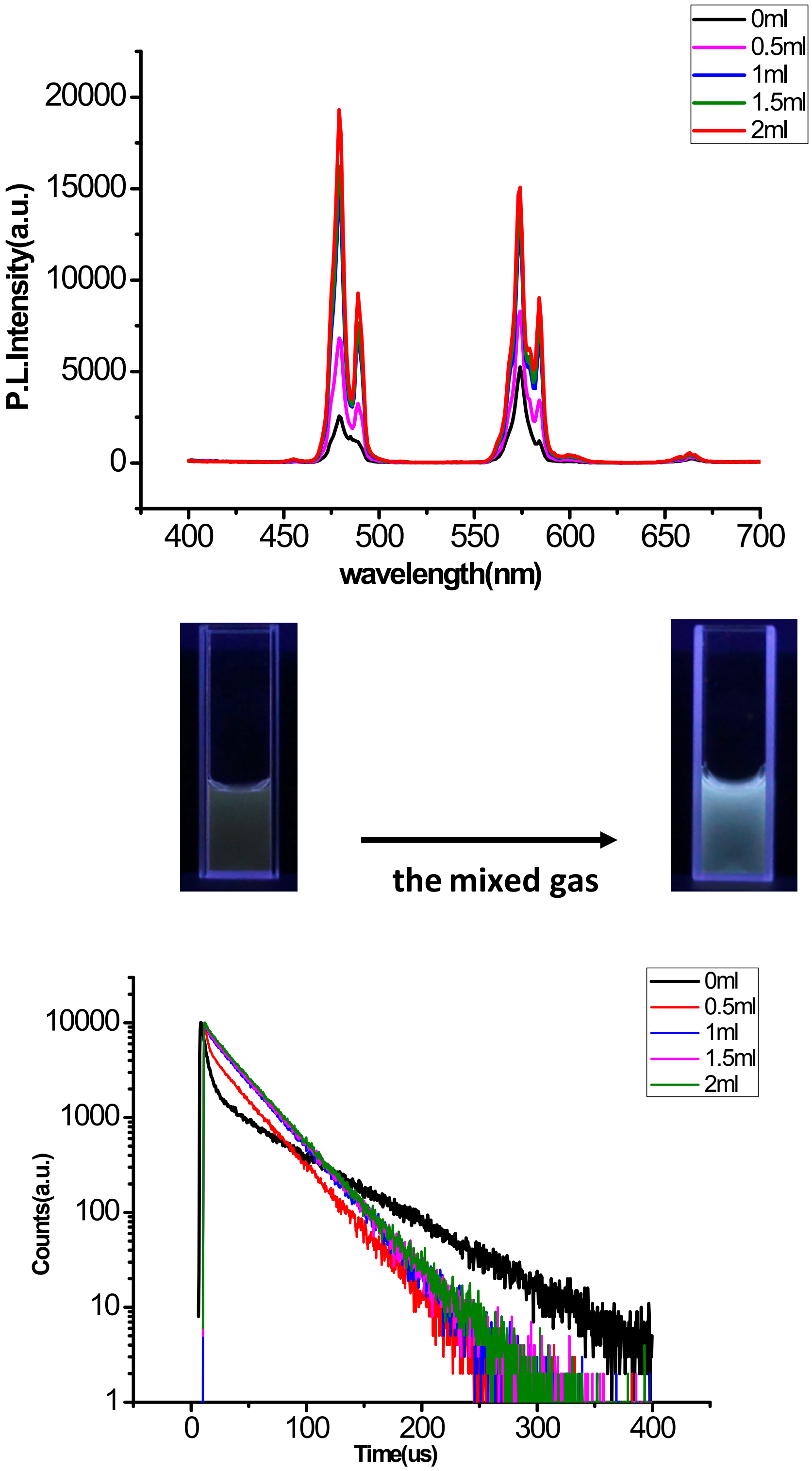

Figure S7. The emission spectra (up) and decay curves (down) of DyW_{10}/ PEO_{113}-*b*-$PDMAEMA_{16}$ complex solution before and after purging the mixed gas (~20% CO_2、70% N_2,10% O_2、0.1% SO_2) for a couple of minutes at 25 °C (λ_{ex} = 280 nm)

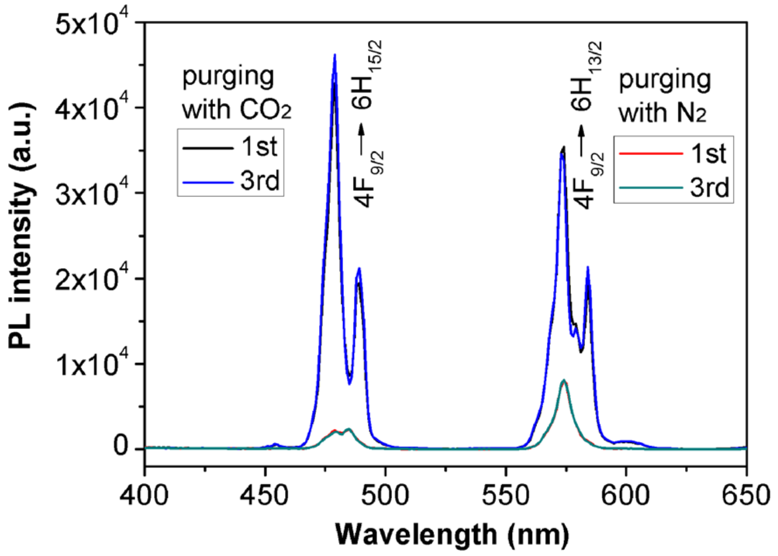

Figure S8. Reversible change in emission spectra (λ_{ex} = 280 nm) of DyW_{10}/PEO-*b*-PDMAEMA complex by alternating CO_2/N_2 treatment.

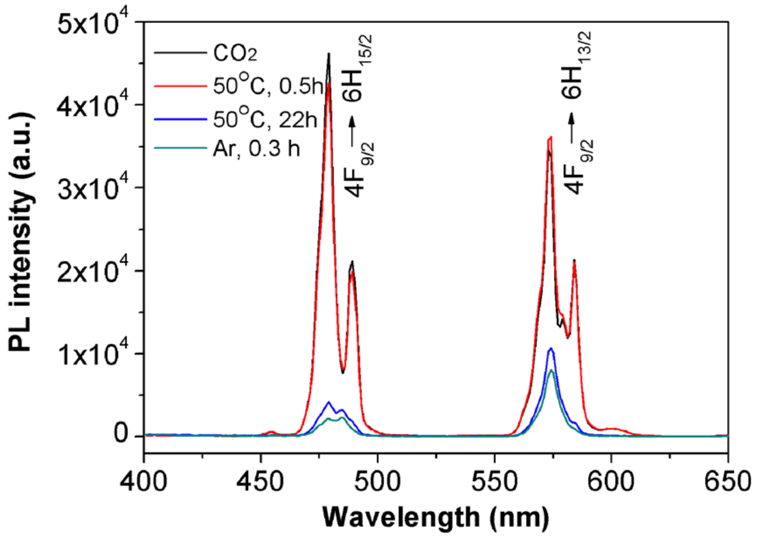

Figure S9. Emission spectra (λ_{ex} =280 nm) of DyW_{10}/PEO-*b*-PDMAEMA hybrid complex after treatment with CO_2, heating, and Ar, respectively.

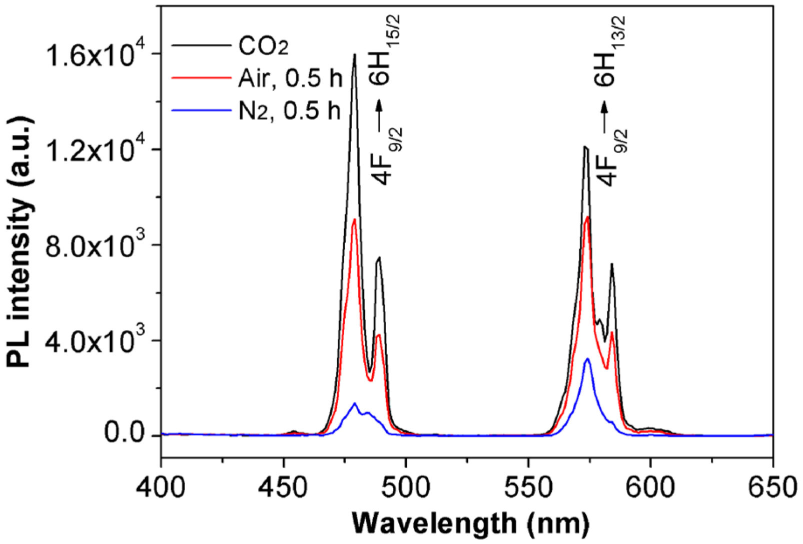

Figure S10. Emission spectra (λ_{ex} =280 nm) of DyW_{10}/PEO-*b*-PDMAEMA hybrid complex after treatment with CO_2, compressed air, and N_2, respectively.

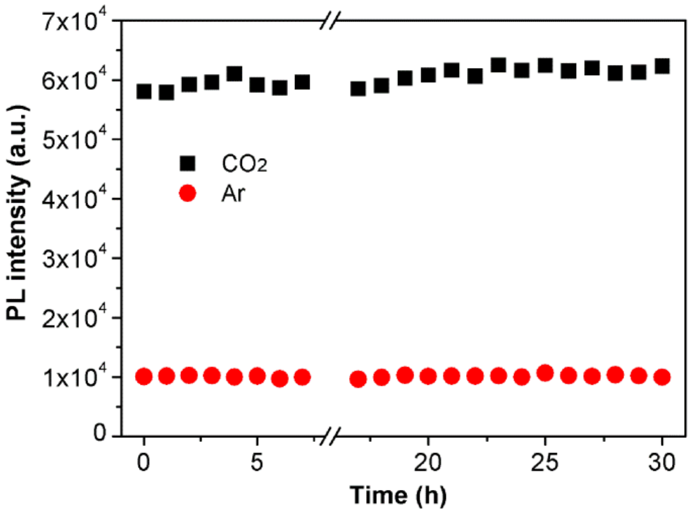

Figure S11. Time dependence of the luminescence intensity for DyW_{10}/PEO-*b*-PDMAEMA solution under the two states at ambient temperature (**CO_2**: upon exposure to CO_2; **Ar**: upon Ar bubbling that removes CO_2).

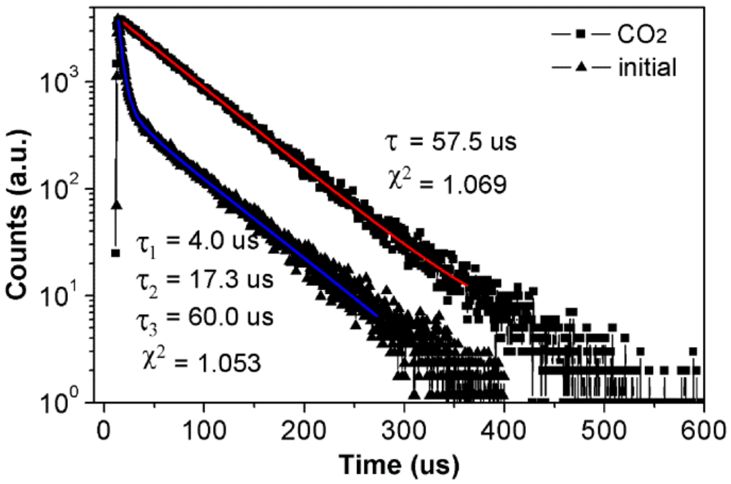

Figure S12. Decay curves of DyW_{10}/PEO-*b*-PDMAEMA coassemblies in aqueous solution before and after CO_2 stimulation.

Table S1. Fitting parameters for luminescence decay curves

Sample	lifetime and its amplitude						
	τ_1 (μs)	f_1	τ_2 (μs)	f_2	τ_3 (μs)	f_3	$\chi^{2\,a}$
DyW_{10} (aq)	5.2	1.000	---	---	---	---	1.150
DyW_{10}/copolymer	4.0	0.366	17.3	0.201	60.0	0.433	1.053
DyW_{10}/copolymer + CO_2	---	---	---	---	57.5	1.000	1.069
DyW_{10}/copolymer[b]	4.4	0.264	20.4	0.164	58.5	0.572	1.039

[a]Chi-squared test of the fitting curve. [b]Obtained by degassed CO_2 dissolved in the solution by purging Ar and slightly warmed to 40 °C.
